# Dietary Resistant Maltodextrin Attenuates Skeletal Muscle Atrophy in Association with Modulation of the Microbiota–Gut–Muscle Axis in Aged Mice

**DOI:** 10.4014/jmb.2606.06037

**Published:** 2026-09-02

**Authors:** Yohan Nam, Hwanju Hwang, Daehyeon Lim, Eunseo Cho, Kiyoung Kim, Soonok Sa, Jung-Sook Han, Wonyong Kim

**Affiliations:** 1 Department of Microbiology, Chung-Ang University College of Medicine, Seoul 06974, Republic of Korea; 2 Food R&D Center, Samyang Corp., Seongnam 13488, Republic of Korea; 3 LuxBiome Co. Ltd., Seoul 06974, Republic of Korea

**Keywords:** Resistant maltodextrin, Prebiotics, Gut microbiota, Sarcopenia, Skeletal muscle atrophy

## Abstract

Resistant maltodextrin (RMD) is a water-soluble dietary fiber with prebiotic properties. This study investigated the effects of RMD on skeletal muscle atrophy and associated molecular and microbial changes in eighteen-month-old mice fed a standard chow diet (SCD) or high-fat diet (HFD) for 12 weeks. RMD supplementation improved muscle strength, endurance, and muscle mass under both dietary conditions. In HFD-fed mice, RMD also reduced fat accumulation and adipocyte size. Gut microbiota analysis showed distinct responses to RMD according to dietary condition, including enrichment of *Lactobacillus* and *Alloprevotella* under SCD and *Allobaculum* and *Akkermansia* under HFD. RMD also increased fecal SCFA levels under HFD conditions and was associated with increased expression of PI3K/Akt/mTOR-related and myogenic genes in skeletal muscle. These findings indicate that RMD attenuates skeletal muscle atrophy in aged mice in association with changes in gut microbiota composition, microbial metabolites, and muscle-related gene expression.

## Introduction

Sarcopenia, characterized by progressive loss of skeletal muscle mass and function with aging, increases the risks of falls, fractures, hospitalization, and mortality [1]. With the rapid aging of the global population, sarcopenia and sarcopenic obesity have emerged as major public health concerns. Sarcopenic obesity is associated with physical inactivity and a higher risk of cardiovascular and metabolic diseases than obesity or sarcopenia alone [2]. Despite its clinical and socioeconomic burden, effective preventive or nutritional strategies remain limited, with regular exercise and balanced dietary intake being the primary recommended interventions.

The gut microbiota plays a critical role in host nutrition and metabolism and exerts systemic effects beyond the gastrointestinal tract, including skeletal muscle. The gut–muscle axis has recently been proposed to describe the influence of gut microbiota composition and microbial metabolites on muscle mass and function. Germ-free mice exhibit marked skeletal muscle atrophy compared with pathogen-free mice, underscoring the importance of microbial colonization in muscle homeostasis [3]. Microbial metabolites, such as short-chain fatty acids (SCFAs) and amino acid–derived compounds, are thought to mediate these effects by modulating host energy metabolism and anabolic signaling pathways [4, 5]. Accordingly, dietary strategies targeting the gut microbiota have gained attention as potential approaches for maintaining muscle health.

Resistant maltodextrin (RMD) is a water-soluble dietary fiber produced by starch hydrolysis and is widely used as a functional food ingredient owing to its prebiotic properties. RMD promotes the growth of beneficial intestinal bacteria and improves postprandial glucose metabolism in both animal and human studies [6-8]. Given the close relationship between obesity and skeletal muscle health [9, 10] and evidence that non-digestible carbohydrates can alter gut microbiota and microbial metabolites [11], RMD-associated microbial changes may also influence muscle homeostasis; however, this possibility has not yet been fully explored.

In this study, we evaluated whether dietary RMD supplementation improves skeletal muscle health in association with alterations in the gut microbiota. Eighteen-month-old mice were fed SCD or HFD with or without RMD supplementation for 12 weeks. We assessed muscle mass, strength, and endurance together with body composition, gut microbiota composition, fecal short-chain fatty acids, and muscle-related gene expression. The inclusion of age-matched SCD groups allowed us to distinguish RMD-associated changes from those associated with HFD feeding and to evaluate the effects of RMD under both standard and high-fat dietary conditions.

## Materials and Methods

### Animals and Experimental Design

**Fig. 1:**
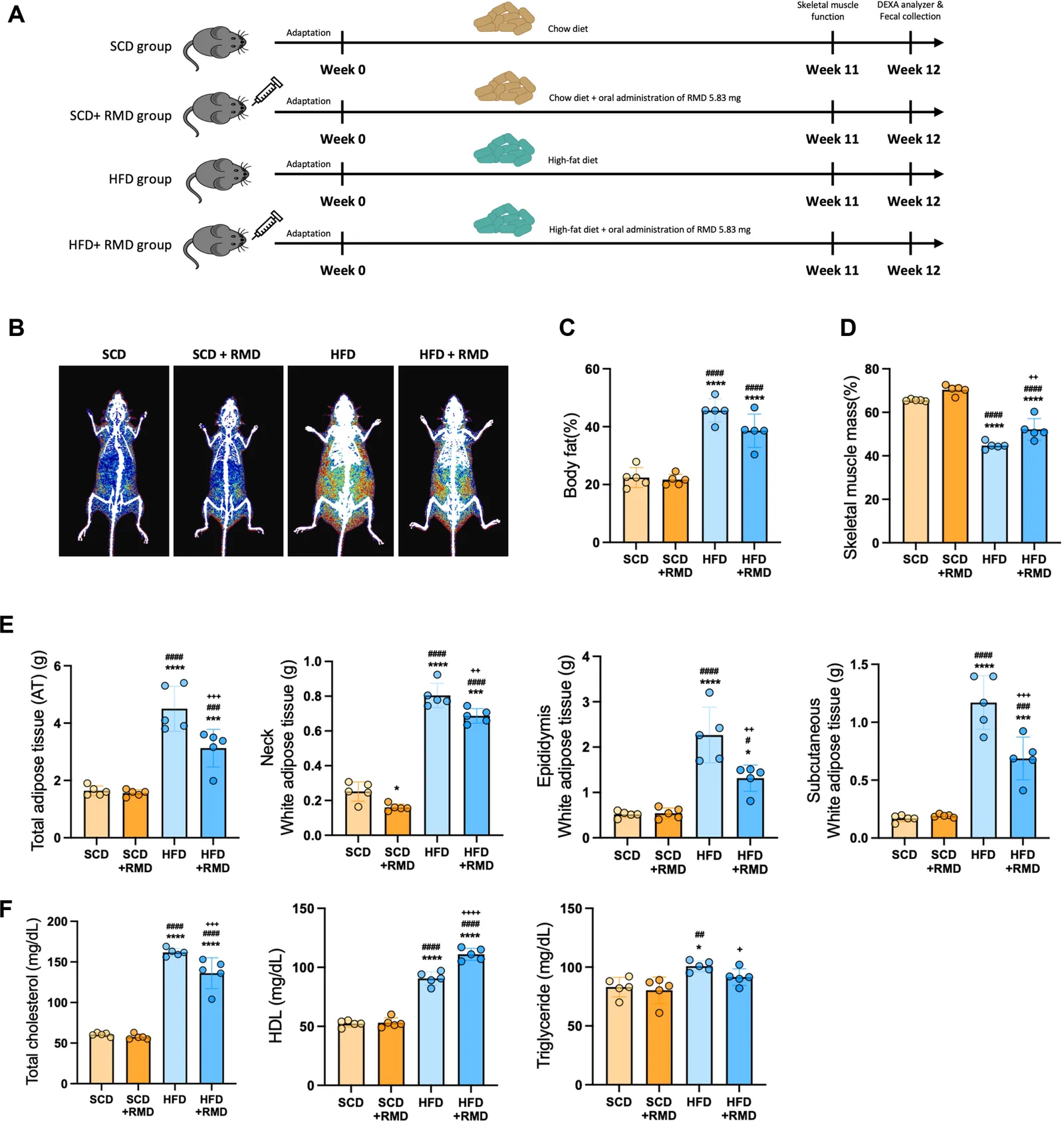
Effects of RMD on body composition and adiposity in aged mice under SCD and HFD conditions. (**A**) Experimental design. (**B**) Representative DEXA images of mice in the four treatment groups. (**C**) Whole-body fat mass and (**D**) whole-body lean mass in the four groups. (**E**) Adipose tissue weights and (**F**) serum lipid levels, including total cholesterol, high-density lipoprotein (HDL) cholesterol, and triglycerides. Data are presented as the mean ± SEM and were analyzed using one-way ANOVA followed by Tukey’s multiple-comparisons test. *P < 0.05, **P < 0.01, ***P < 0.001, ****P < 0.0001 vs. SCD group; ^#^*P* < 0.05, ^##^*P* < 0.01, ^###^*P* < 0.001, ^####^*P* < 0.0001 vs. SCD+RMD group; ^+^*P* < 0.05, ^++^*P* < 0.01, ^+++^*P* < 0.001, ^++++^*P* < 0.0001 vs. HFD group.

Eighteen-month-old male C57BL/6J mice (RaonBio Co., Ltd., Republic of Korea) were acclimated for 1 week under controlled conditions (23 ± 1°C, 50 ± 5% relative humidity, 12-h light/dark cycle, and 10–12 air changes per h). Mice were randomly assigned to four groups (n = 6/group): SCD, SCD+RMD, HFD, and HFD+RMD. Mice were fed either a standard chow diet (SCD; Rodent NIH-41; Zeigler Feeds, USA) or a high-fat diet (HFD; Rodent Diet With 60% kcal Fat; Research Diets, USA) for 12 weeks. In the RMD-treated groups, resistant maltodextrin (Fiberest®, Samyang Co., Republic of Korea) was orally administered at 5.83 mg/day for 12 weeks, based on a human intake of 10 g/day for a 60-kg adult and a mouse body weight of 35 g. Mice had ad libitum access to water, and food intake was controlled by pair-feeding within each dietary condition and monitored weekly. At the end of the study, mice were anesthetized with isoflurane (2–3% in oxygen) and euthanized by CO_2_ inhalation followed by cervical dislocation. Tissue and blood samples were collected and stored in formalin or liquid nitrogen until analysis. The experimental design is shown in Fig. 1A. All procedures were approved by the Institutional Animal Care and Use Committee of Chung-Ang University (IACUC No. A2022067) and conducted in accordance with relevant guidelines, including the ARRIVE guidelines.

### Muscular Strength and Endurance Tests

Muscular strength was assessed using the four-limb hanging test. Each mouse was placed on a wire mesh (approximately 30 × 30 cm; wire diameter < 0.5 cm), which was then inverted. The hanging time (in seconds) was recorded as an indicator of muscle strength. Measurements were performed at 2-week intervals, for a total of three trials, with a resting period of at least 30 min between trials to minimize fatigue. Muscular endurance was evaluated using a motorized six-lane treadmill (Jeong Do B&P, Republic of Korea) at 2-week intervals. Prior to testing, mice were acclimated to the treadmill at a speed of 10 m/min with a 0° incline and motivated by exposure to a mild electrical stimulus delivered through the grid. The endurance test was initiated at 8 m/min with a 0° incline, followed by incremental increases of 2 m/min in speed and 2° in incline every 5 min. Muscular endurance was defined as the total running distance covered until the mouse remained on the grid for more than 15 s without re-entering the running lane.

### Body Composition and Skeletal Muscle Mass Measurements

*In vivo* body composition, including whole-body fat mass and lean mass, was assessed in live mice using dual-energy X-ray absorptiometry (DEXA) with an InAlyzer system (Medikors, Republic of Korea). This non-invasive imaging technique enabled quantitative evaluation of whole-body fat and lean mass. Following sacrifice, hind limb skeletal muscles, specifically the gastrocnemius (GAS) and soleus (SOL), were carefully excised and weighed to validate the *in vivo* DEXA measurements and to assess skeletal muscle atrophy. Relative muscle weight was calculated as individual muscle weight normalized to body weight, as previously described [12].

### RT-qPCR

Total RNA was extracted from gastrocnemius (GAS), subcutaneous white adipose tissue, and ileum samples using the RNeasy Mini Kit (Qiagen, Germany) according to the manufacturer’s instructions. RNA concentration and purity were measured using a NanoQuant spectrophotometer (Infinite M200; Tecan, Switzerland). Complementary DNA (cDNA) was synthesized from 1 ng of total RNA according to the manufacturer’s recommendations using the PrimeScript 1st Strand cDNA Synthesis Kit (Takara Bio, Japan). Quantitative PCR was performed using SYBR Green Master Mix on a QuantStudio 3 real-time PCR system (Applied Biosystems, USA). The PCR cycling conditions consisted of an initial denaturation at 95°C for 30 s, followed by 40 cycles of denaturation at 95°C for 5 s and annealing/extension at 60°C for 30 s. Relative mRNA expression levels were calculated using the 2^–ΔΔCt^ method, with β-actin used as an internal control for normalization. Primer sequences used for RT-qPCR are listed in Tables S1 and S2.

### Biochemical Analyses

Blood samples were collected via the retro-orbital vein and allowed to clot at room temperature (25–27°C) for 1 h. Samples were then centrifuged at 5,000 × g for 1 h to obtain serum, which was stored at -80°C until analysis. Serum levels of total cholesterol, high-density lipoprotein (HDL) cholesterol, and triglycerides were measured by Seoul Clinical Laboratories (SCL Healthcare, Republic of Korea) using standardized enzymatic assay methods.

### Histological Analysis

Gastrocnemius muscle and white and brown adipose tissues were fixed in 4% paraformaldehyde, followed by paraffin embedding. Sections were stained with hematoxylin and eosin (H&E) to assess overall muscle morphology. Histological images were captured using a light microscope at 200 magnification. Quantitative analyses of muscle fiber cross-sectional area (CSA), muscle morphology, and adipocyte area were performed using ImageJ software (National Institutes of Health, USA). For each sample, five randomly selected fields were analyzed to obtain representative measurements.

### Microbiome Sequencing

To assess differences in gut microbiota composition among the experimental groups (SCD, SCD+RMD, HFD, and HFD+RMD), fecal samples were collected from each mouse at week 12 and stored at -80°C until analysis. Total genomic DNA was extracted from fecal samples using the FastDNA Spin Kit (MP Biomedicals, USA) according to the manufacturer’s instructions. Full-length 16S rRNA gene libraries were generated by PCR amplification and sequenced using the PacBio Sequel IIe platform (Pacific Biosciences, USA) in long-read HiFi mode. Amplicon sequence variants (ASVs) were inferred using the DADA2 pipeline implemented in R (version 4.0.2) and taxonomically assigned using the SILVA reference database (version 138.1) [13].

Microbiome data analyses were performed using the phyloseq, vegan, tidyverse, and DESeq2 packages [14-17]. Alpha diversity was evaluated using the Chao1 and Shannon indices, while beta diversity was assessed by principal coordinates analysis (PCoA) based on Bray–Curtis dissimilarity. Differentially abundant taxa were identified using linear discriminant analysis effect size (LEfSe), with thresholds of an LDA score > 2 and *P* < 0.001. Correlations between gut microbial genera and muscle-related parameters were examined using Spearman’s correlation analysis, with associations considered significant at *P* < 0.05. Functional profiles of the intestinal microbiota were predicted using PICRUSt2 and visualized using STAMP software (version 2.1.3).

### SCFA Analysis

At week 12, fecal samples (100 mg) were collected from each group (SCD, SCD+RMD, HFD, and HFD+RMD) and stored at -80°C until analysis. Samples were homogenized in 1 mL of distilled water and centrifuged at 10,000 × g for 15 min at 4°C. The resulting supernatants were filtered using Sep-Pak Vac cartridges (WAT054955; Waters, USA). SCFAs were separated using an Aminex 87H column (300 × 10 mm; Bio-Rad, USA) with an isocratic 0.01 N H_2_SO_4_ mobile phase (Fluka, USA) at a flow rate of 0.5 mL/min and a column temperature of 40°C and were detected using a refractive index detector (RefractoMAX520; ERC, Japan). Acetic, propionic, and butyric acids were identified by matching retention times with the corresponding authentic standards (VOA mixture, 10 mM; AccuStandard FAMQ-004, USA) and were quantified using calibration curves generated from these standards.

### Statistical Analysis

All data are presented as the mean ± standard error of the mean (SEM). Statistical analyses and data visualization were performed using GraphPad Prism software (version 9.1; GraphPad Software, USA). Longitudinal measurements of muscle strength and endurance were analyzed using two-way repeated-measures ANOVA with group and time as factors, followed by Šidák’s multiple-comparisons test. Other physiological, biochemical, histological, gene-expression, and SCFA data were analyzed using one-way ANOVA followed by Tukey’s multiple-comparisons test. Correlations between gut microbial genera and muscle-related parameters were assessed using Spearman’s rank correlation analysis. Pairwise comparisons of predicted microbial functional profiles were performed using the Wilcoxon rank-sum test. A *P* value < 0.05 was considered statistically significant.

## Results

### RMD Supplementation Differentially Affects Body Composition under SCD and HFD Conditions

To evaluate the effects of resistant maltodextrin (RMD) on body composition in aged mice, eighteen-month-old mice were fed SCD or HFD with or without RMD supplementation for 12 weeks (Fig. 1A). Whole-body composition was assessed *in vivo* using DEXA.

Whole-body fat mass did not differ significantly between the SCD and SCD+RMD groups. In contrast, HFD-fed mice exhibited greater fat accumulation than SCD-fed mice, and the HFD group showed lower skeletal muscle mass than the SCD groups (Fig. 1B–1D). RMD supplementation under HFD conditions was associated with lower whole-body fat mass and greater whole-body lean mass than in the HFD group, whereas the body-composition effect of RMD was less pronounced under SCD conditions.

Consistent with the DEXA findings, total adipose tissue depot weight was higher in HFD-fed mice than in SCD-fed mice and was lower in the HFD+RMD group than in the HFD group (Fig. 1E). In the HFD comparison, RMD supplementation was associated with approximately 15.25 ± 12.61% lower whole-body fat mass, approximately 16.56 ± 11.11% greater whole-body lean mass than in the HFD group, and approximately 30.55 ± 14.60% lower total adipose tissue depot weight. Serum total cholesterol and triglyceride levels were also lower, whereas HDL cholesterol was higher, in the HFD+RMD group than in the HFD group (Fig. 1F).

### RMD Supplementation Reduces Adiposity and Increases Thermogenesis-Related Gene Expression Primarily under HFD Conditions

**Fig. 2:**
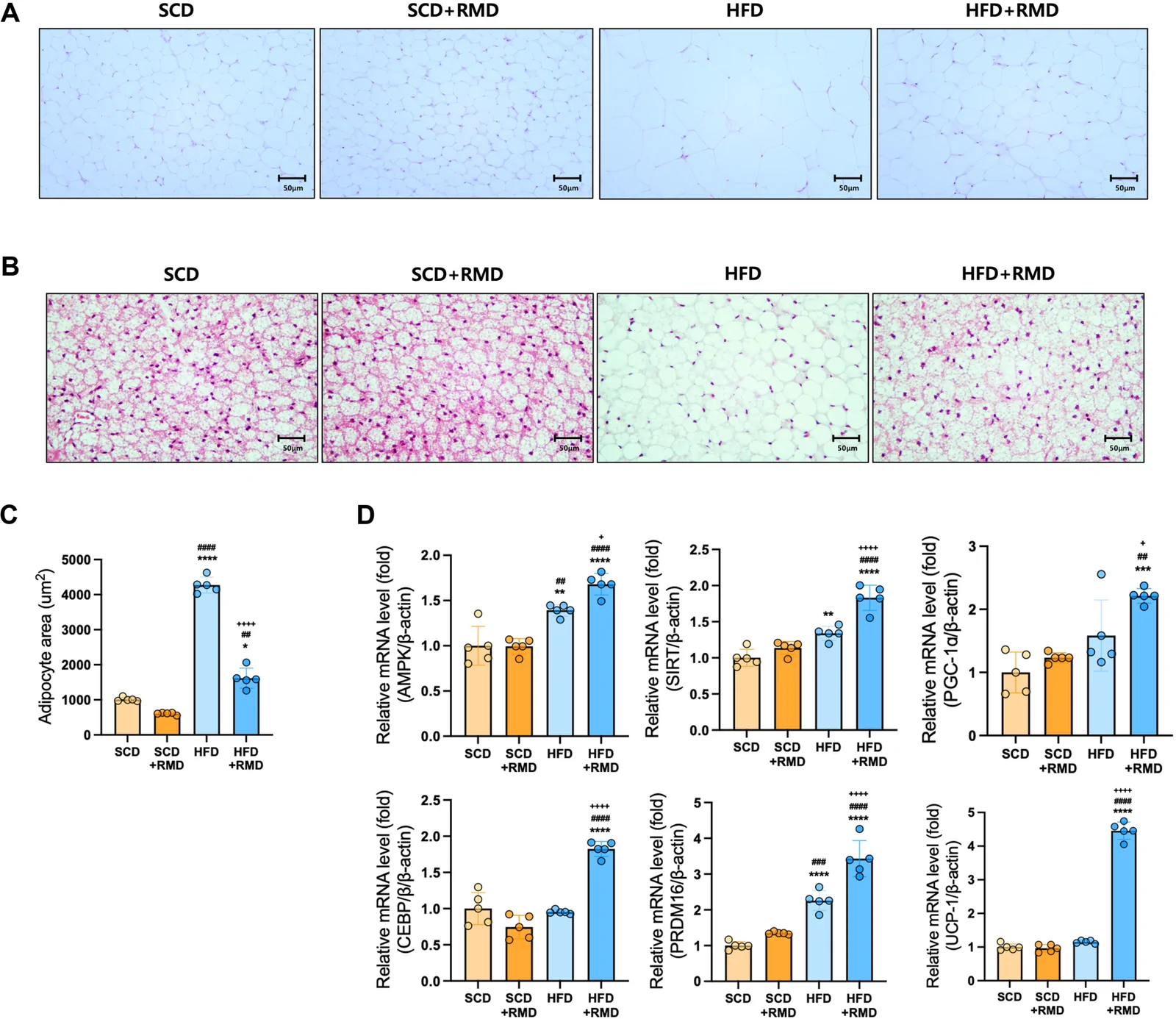
Effects of RMD on adipose tissue morphology and thermogenesis-related gene expression in aged mice. (**A**) Hematoxylin and eosin staining of subcutaneous white adipose tissue and (**B**) brown adipose tissue from the four treatment groups. (**C**) Adipocyte area in subcutaneous white adipose tissue. (**D**) mRNA expression of AMPK, SIRT1, PGC-1α, C/EBPβ, PRDM16, and UCP1 in subcutaneous white adipose tissue. Data are presented as the mean ± SEM and were analyzed using one-way ANOVA followed by Tukey’s multiple-comparisons test. *P < 0.05, **P < 0.01, ***P < 0.001, ****P < 0.0001 vs. SCD group; ^#^*P* < 0.05, ^##^*P* < 0.01, ^###^*P* < 0.001, ^####^*P* < 0.0001 vs. SCD+RMD group; ^+^*P* < 0.05, ^++^*P* < 0.01, ^+++^*P* < 0.001, ^++++^*P* < 0.0001 vs. HFD group.

Histological analyses of white and brown adipose tissues were performed to characterize diet- and RMD-associated adipose changes (Fig. 2A and 2B). Adipocyte size was greater in HFD-fed mice than in SCD-fed mice. RMD supplementation reduced adipocyte size under both dietary conditions, with a significant reduction in the HFD+RMD group compared with the HFD group (Fig. 2C).

To determine whether these morphological differences were accompanied by changes in thermogenesis-related gene expression, subcutaneous white adipose tissue was analyzed by quantitative reverse transcription PCR (Fig. 2D). RMD supplementation did not significantly alter the expression of the examined thermogenesis-related genes under SCD conditions. In contrast, the mRNA levels of AMP-activated protein kinase (AMPK), sirtuin 1 (SIRT1), peroxisome proliferator-activated receptor γ coactivator 1α (PGC-1α), CCAAT/enhancer-binding protein β (C/EBPβ), PR domain-containing protein 16 (PRDM16), and uncoupling protein 1 (UCP1) were significantly higher in the HFD+RMD group than in the HFD group.

In the HFD comparison, mean adipocyte cross-sectional area in white adipose tissue was approximately 62.12 ± 6.70% lower in the HFD+RMD group than in the HFD group (Fig. 2C). This difference was accompanied by 1.21 ± 0.08- to 3.87 ± 0.22-fold higher mRNA expression of thermogenesis-related genes in RMD-supplemented mice (Fig. 2D).

### RMD Supplementation Mitigates Skeletal Muscle Atrophy and Improves Muscle Function

**Fig. 3:**
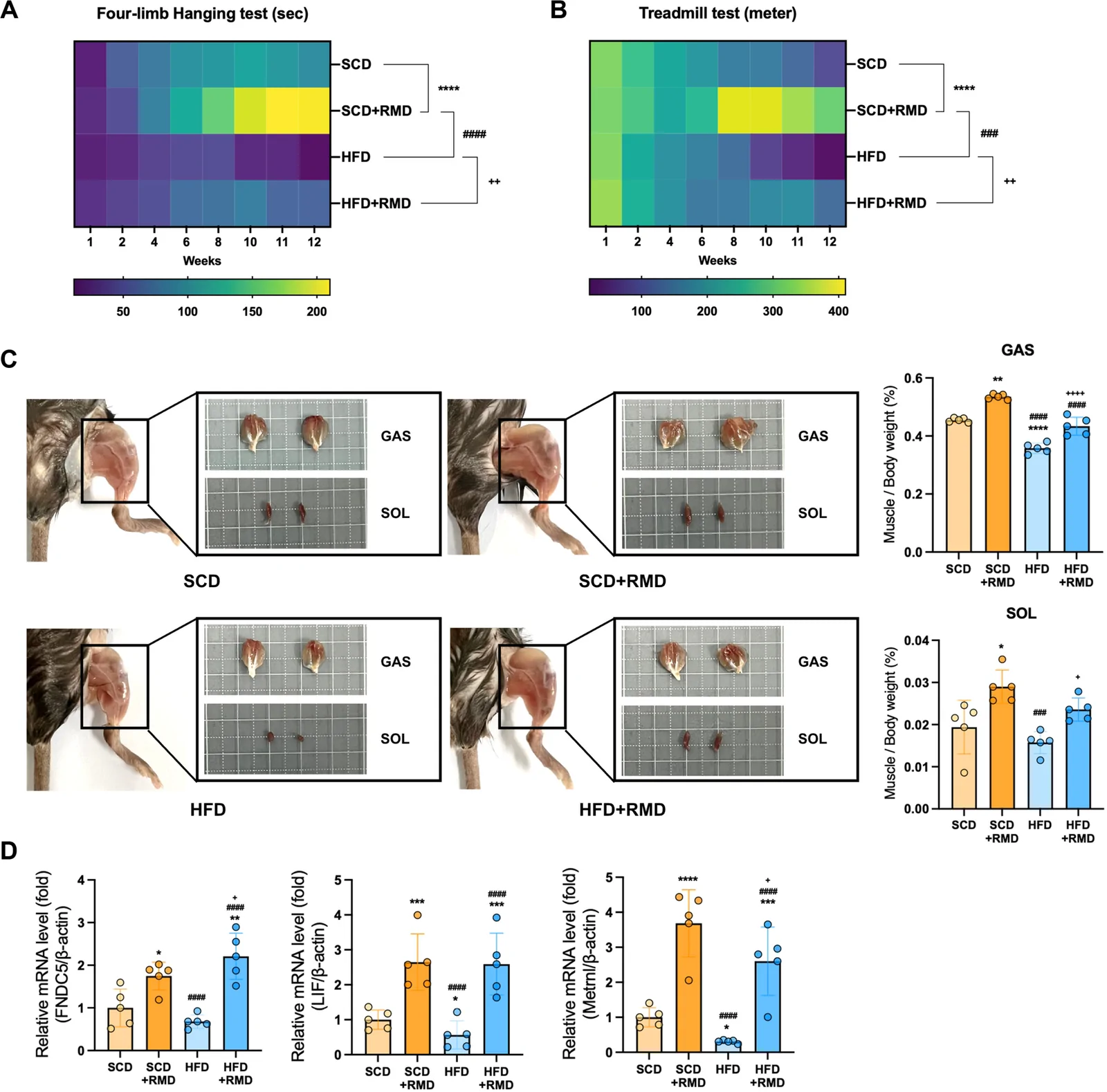
Effects of RMD on skeletal muscle mass and function in aged mice. (**A**) Four-limb hanging test and (**B**) treadmill endurance test during the 12-week intervention. (**C**) Representative gastrocnemius (GAS) and soleus (SOL) muscles and their weights at the end of the study. (**D**) mRNA expression of FNDC5, LIF, and Metrnl in skeletal muscle. Data are presented as the mean ± SEM. Longitudinal measurements in panels A and B were analyzed using two-way repeated-measures ANOVA with group and time as factors, followed by Šidák’s multiple-comparisons test. Data in panels C and D were analyzed using one-way ANOVA followed by Tukey’s multiple-comparisons test. *P < 0.05, **P < 0.01, ***P < 0.001, ****P < 0.0001 vs. SCD group; ^#^*P* < 0.05, ^##^*P* < 0.01, ^###^*P* < 0.001, ^####^*P* < 0.0001 vs. SCD+RMD group; ^+^*P* < 0.05, ^++^*P* < 0.01, ^+++^*P* < 0.001, ^++++^*P* < 0.0001 vs. HFD group.

Muscular strength was evaluated using the four-limb hanging test during the 12-week experimental period. RMD supplementation significantly improved hanging performance under both dietary conditions, with differences evident from week 9 in the SCD+RMD group and from week 5 in the HFD+RMD group compared with their respective non-supplemented controls (Fig. 3A). Treadmill endurance was also significantly greater in both RMD-supplemented groups, with differences evident from week 7 under SCD conditions and from week 9 under HFD conditions (Fig. 3B).

Consistent with these functional improvements, gastrocnemius (GAS) and soleus (SOL) muscle weights were significantly greater in the RMD-supplemented groups than in their respective diet controls (Fig. 3C). Muscle weights were lower in HFD-fed mice than in SCD-fed mice, indicating an additional adverse effect of HFD on skeletal muscle in aged mice. To characterize molecular changes associated with muscle function and energy metabolism, the expression of the myokines fibronectin type III domain-containing protein 5 (FNDC5), leukemia inhibitory factor (LIF), and meteorin-like protein (Metrnl) was analyzed in skeletal muscle (Fig. 3D).

The mRNA levels of these myokines were lower in HFD-fed mice than in SCD-fed mice and were significantly higher following RMD supplementation under both dietary conditions. Metrnl expression was particularly high in the SCD+RMD group compared with the HFD+RMD group.

Within the HFD comparison, RMD supplementation increased four-limb hanging time by approximately 2.25 ± 1.34-fold and treadmill running distance by approximately 117.79 ± 189.38% compared with HFD-fed aged mice (Fig. 3A and 3B). Gastrocnemius and soleus muscle weights were also higher by approximately 21.30 ± 8.68% and 49.80 ± 17.49%, respectively, in the HFD+RMD group (Fig. 3C), consistent with attenuation of HFD-associated skeletal muscle atrophy.

### RMD Supplementation Increases Muscle Fiber Size and Anabolic Signaling-Related Gene Expression in Skeletal Muscle

**Fig. 4:**
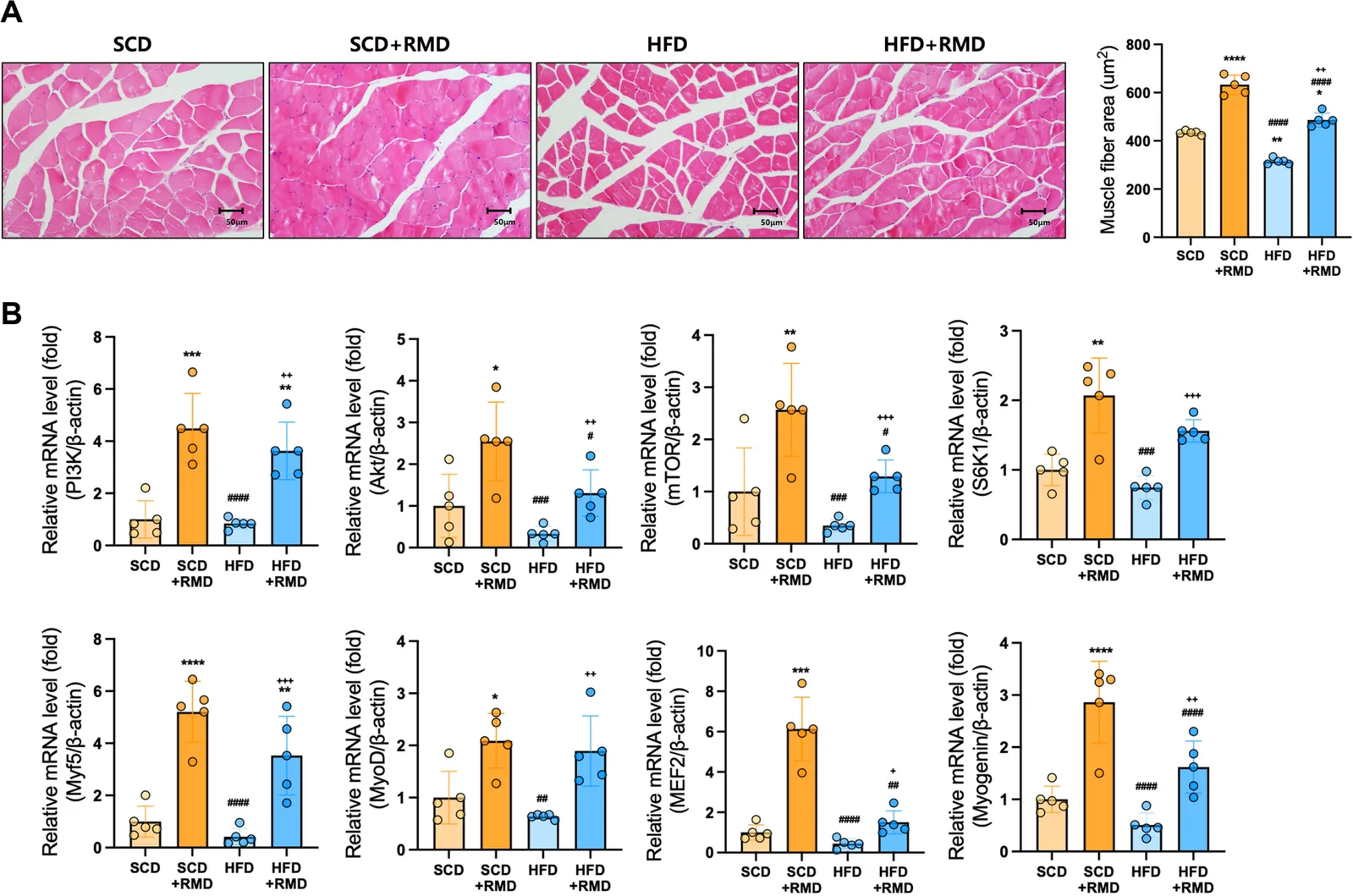
Effects of RMD on skeletal muscle fiber size and muscle-related gene expression in aged mice. (**A**) Hematoxylin and eosin staining of gastrocnemius muscle and quantification of muscle fiber cross-sectional area in the four treatment groups. (**B**) mRNA expression of PI3K, Akt, mTOR, S6K1, Myf5, MyoD, MEF2, and myogenin in skeletal muscle. Data are presented as the mean ± SEM and were analyzed using one-way ANOVA followed by Tukey’s multiple-comparisons test. *P < 0.05, **P < 0.01, ***P < 0.001, ****P < 0.0001 vs. SCD group; ^#^*P* < 0.05, ^##^*P* < 0.01, ^###^*P* < 0.001, ^####^*P* < 0.0001 vs. SCD+RMD group; ^+^*P* < 0.05, ^++^*P* < 0.01, ^+++^*P* < 0.001, ^++++^*P* < 0.0001 vs. HFD group.

The effects of resistant maltodextrin (RMD) supplementation on skeletal muscle morphology and anabolic signaling-related gene expression were evaluated in the gastrocnemius muscle of aged mice under SCD and HFD conditions. Gastrocnemius muscle fiber cross-sectional area (CSA) was lower in the HFD group than in the SCD group and was significantly greater following RMD supplementation under both dietary conditions (Fig. 4A).

To examine molecular changes associated with these morphological differences, we measured the mRNA expression of key components of the PI3K/Akt/mTOR pathway and myogenesis-related transcription factors. The mRNA levels of phosphoinositide 3-kinase (PI3K), protein kinase B (Akt), mechanistic target of rapamycin (mTOR), and S6 kinase 1 (S6K1) were lower in the HFD group than in the SCD group and were significantly higher in the RMD-supplemented groups than in their corresponding diet controls (Fig. 4B). Similarly, mRNA expression of Myf5, MyoD, myocyte enhancer factor 2 (MEF2), and myogenin was higher following RMD supplementation, with particularly pronounced changes under SCD conditions (Fig. 4B).

Within the HFD comparison, mean gastrocnemius muscle fiber CSA was approximately 54.05 ± 7.77% greater in the HFD+RMD group than in the HFD group. This morphological difference was accompanied by approximately 2.09 ± 0.22- to 4.32 ± 1.31-fold higher mRNA expression of PI3K, Akt, mTOR, and S6K1 in RMD-supplemented mice (Fig. 4A and 4B).

### RMD Supplementation Alters Gut Microbiota Composition under SCD and HFD Conditions and Is Associated with Muscle-Related Factors

**Fig. 5:**
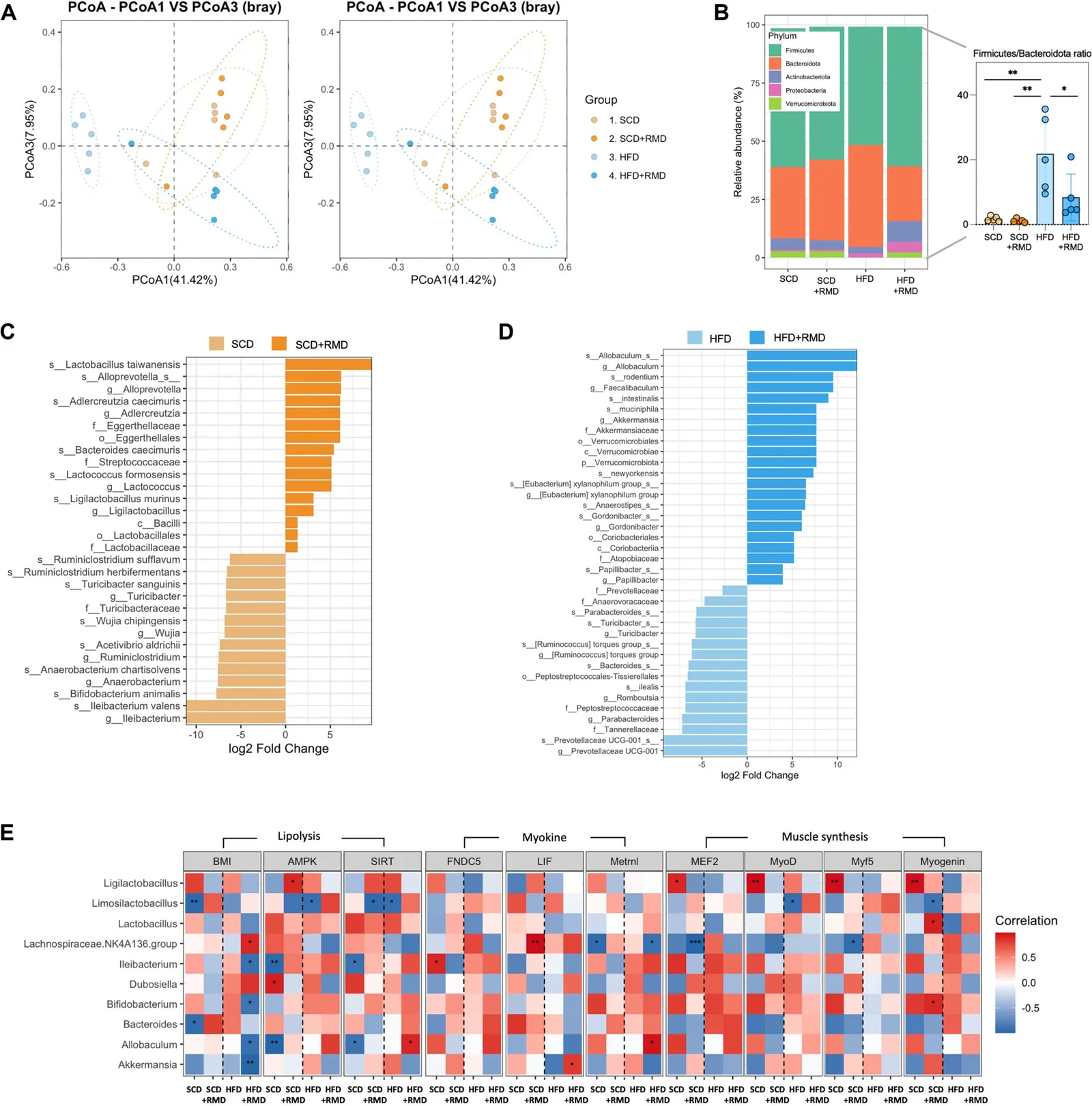
Effects of RMD on the fecal microbiota under SCD and HFD conditions in aged mice. (**A**) Principal coordinates analysis (PCoA) based on Bray–Curtis dissimilarity. (**B**) Relative abundance of major bacterial phyla and the Firmicutes/Bacteroidota ratio. (**C**) LEfSe analysis comparing the SCD and SCD+RMD groups. (**D**) LEfSe analysis comparing the HFD and HFD+RMD groups. (**E**) Heatmap of Spearman’s correlations between discriminatory microbial genera and muscle-related or metabolic parameters. The color scale indicates the correlation coefficient, with red and blue representing positive and negative correlations, respectively. Significant correlations are indicated in the figure as *P < 0.05, **P < 0.01, ***P < 0.001, and ****P < 0.0001.

To determine whether the gut microbial response to RMD differed according to dietary context, fecal samples from all four groups were analyzed using 16S rRNA gene sequencing. Alpha diversity indices, including Chao1 richness and Shannon diversity, did not differ significantly between SCD and SCD+RMD or between HFD and HFD+RMD groups (Fig. S1). Principal coordinates analysis (PCoA) based on Bray–Curtis dissimilarity showed distinct positioning of the HFD group, whereas HFD+RMD samples were positioned closer to SCD-fed groups in ordination space (Fig. 5A). At the phylum level, the HFD+RMD group also showed a lower Firmicutes/Bacteroidota ratio than the HFD group (Fig. 5B).

LEfSe analysis demonstrated distinct RMD-associated taxa under the two dietary conditions (Fig. 5C and 5D). Under SCD conditions, RMD supplementation was associated with enrichment of Lactobacillaceae/Lactobacillus-related taxa and *Alloprevotella*. Under HFD conditions, RMD supplementation was associated with enrichment of *Akkermansia muciniphila*, *Allobaculum*, *Faecalibaculum rodentium*, and related taxa, whereas Bacteroides, *Turicibacter*, and the *Ruminococcus torques* group were more abundant in the HFD group. Thus, the taxa associated with RMD supplementation differed substantially between SCD and HFD conditions.

Correlation analysis showed associations between microbial taxa and muscle-related parameters under the two dietary conditions (Fig. 5E). In the SCD+RMD group, Lactobacillus abundance was positively associated with myogenin expression. In the HFD+RMD group, *Akkermansia* abundance was positively associated with leukemia inhibitory factor (LIF) expression.

### RMD Supplementation Is Associated with Changes in Predicted Microbial Functions and SCFA Profiles under SCD and HFD Conditions

**Fig. 6:**
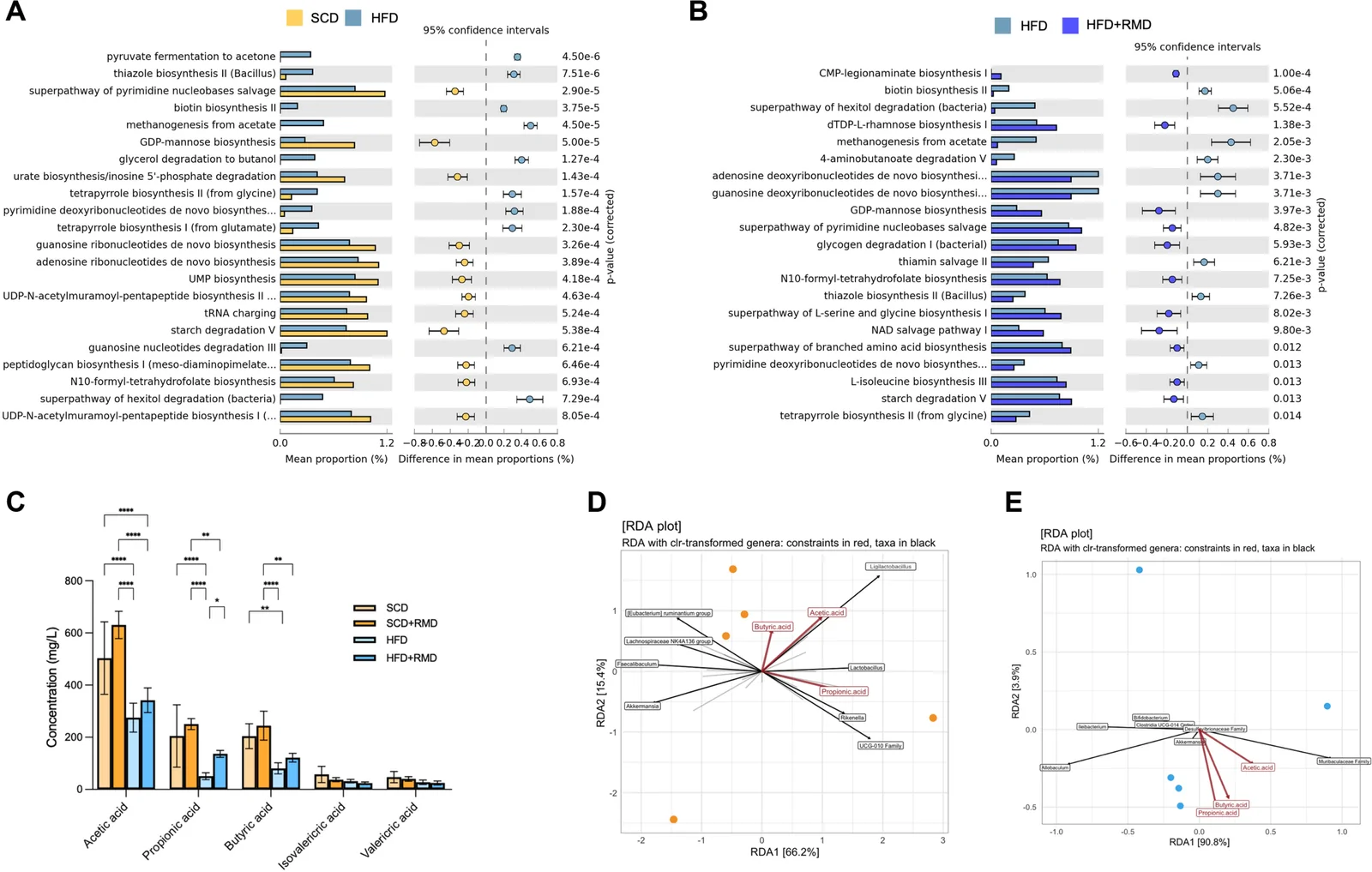
RMD-associated changes in predicted microbial functions and fecal SCFA profiles under SCD and HFD conditions in aged mice. (**A**) Predicted functional differences between the SCD and HFD groups. (**B**) Predicted functional differences between the HFD and HFD+RMD groups. (**C**) Fecal acetate, propionate, and butyrate levels in the four treatment groups. (**D, E**) Redundancy analysis (RDA) showing associations between SCFAs and dominant microbial taxa under SCD (**D**) and HFD (**E**) conditions. Data are presented as the mean ± SEM. Pairwise comparisons of predicted microbial functional profiles in panels A and B were performed using the Wilcoxon rank-sum test. SCFA levels in panel C were analyzed using one-way ANOVA followed by Tukey’s multiple-comparisons test. Significant associations in panels D and E are indicated in the figure as *P < 0.05, **P < 0.01, and ***P < 0.001.

Functional profiles of the intestinal microbiota were predicted using PICRUSt2. Comparisons between SCD- and HFD-fed aged mice showed diet-associated differences in predicted microbial metabolic pathways, including pathways related to fermentation, biotin biosynthesis, and methanogenesis from acetate (Fig. 6A). RMD supplementation was also associated with distinct changes in predicted microbial functions under SCD and HFD conditions. Under HFD conditions, pathways related to amino acid and carbohydrate metabolism, including nucleotide and sugar biosynthesis, glycogen and starch degradation, and branched-chain amino acid biosynthesis, were enriched in the HFD+RMD group compared with the HFD group (Fig. 6B). RMD-associated functional changes under SCD conditions are shown in Fig. S2.

Fecal SCFAs were measured to assess diet- and RMD-associated differences in microbial metabolites. Acetate, propionate, and butyrate levels were lower in HFD-fed mice than in SCD-fed mice (Fig. 6C). RMD supplementation increased SCFA levels, with significant increases observed in the HFD+RMD group compared with the HFD group. Redundancy analysis (RDA) showed diet-specific associations between microbial taxa and SCFAs: under SCD+RMD conditions, *Ligilactobacillus* was positively associated with acetate, whereas under HFD+RMD conditions, *Akkermansia* and members of the Desulfovibrionaceae family were positively associated with propionate and butyrate (Fig. 6D and 6E).

Within the HFD comparison, fecal acetate, propionate, and butyrate levels were higher by approximately 9.97 ± 15.17%, 95.41 ± 55.69%, and 31.30 ± 17.88%, respectively, in RMD-supplemented mice. These metabolite differences occurred in parallel with changes in predicted microbial metabolic pathways and microbial composition.

## Discussion

Despite advances in clinical research, effective nutritional strategies for attenuating age-related skeletal muscle loss remain limited. Resistant maltodextrin (RMD) is a soluble dietary fiber with prebiotic properties and reported benefits for bowel function and glycemic control [7, 8, 18]. In the present study, RMD supplementation improved muscle strength, endurance, and muscle mass in aged mice under both SCD and HFD conditions, while its effects on adiposity, gut microbiota composition, and fecal short-chain fatty acids (SCFAs) differed between the two dietary conditions. These findings support an association between RMD intake and coordinated muscle, metabolic, and microbial changes without establishing a causal microbiota–gut–muscle mechanism.

In HFD-fed aged mice, adipocyte hypertrophy and metabolic dysfunction may contribute to impaired muscle homeostasis [2, 19]. RMD supplementation reduced adiposity and adipocyte size and improved serum lipid profiles under HFD conditions. These metabolic improvements may have contributed indirectly to skeletal muscle preservation [9, 19, 20]. However, RMD also improved muscle strength, endurance, and gastrocnemius and soleus muscle weights under SCD conditions, despite no significant difference in whole-body fat mass between the SCD and SCD+RMD groups. Thus, reduced adiposity is unlikely to fully account for the muscle benefits associated with RMD, although improvements in adiposity and systemic metabolic status may contribute to these effects under HFD conditions.

The PI3K/Akt/mTOR pathway plays an important role in the regulation of skeletal muscle mass and growth [21–23]. RMD supplementation was associated with higher mRNA expression of PI3K, Akt, mTOR, S6K1, and myogenic transcription factors under both dietary conditions. In contrast, no consistent differences were observed in the expression of the muscle atrophy-related genes FOXO3a, atrogin-1, and MuRF-1. Given the multifactorial regulation of age-related muscle loss [24], these findings are best interpreted as changes in anabolic- and myogenesis-related gene expression rather than evidence of activation of a specific signaling pathway. Importantly, PI3K/Akt signaling was assessed only at the mRNA level. Protein-level analyses, including phosphorylation of relevant pathway components, will therefore be required to establish whether RMD alters pathway activity.

Growing evidence supports a relationship between the gut microbiota and skeletal muscle mass and function during aging [3, 25, 26], while HFD-associated obesity has been linked to alterations in gut microbiota composition and impaired skeletal muscle growth signaling [27]. In the present four-group comparison, the microbial response to RMD differed between SCD and HFD conditions. RMD supplementation was associated with enrichment of Lactobacillus-related taxa and *Alloprevotella* under SCD conditions, whereas *Akkermansia* and *Allobaculum* were enriched under HFD conditions. The HFD+RMD group was also positioned closer to the SCD-fed groups than the HFD group in PCoA ordination space and showed a lower Firmicutes/Bacteroidota ratio. In addition, *Akkermansia* abundance was positively associated with LIF expression under HFD conditions, whereas *Lactobacillus* abundance was positively associated with myogenin expression under SCD conditions. These findings indicate that RMD-associated microbial changes vary according to dietary condition. Although the HFD+RMD microbial profile showed features closer to those of SCD-fed mice, the endpoint design does not establish that RMD reverses HFD-induced dysbiosis.

Fecal acetate, propionate, and butyrate levels were lower in HFD-fed mice than in SCD-fed mice, whereas RMD supplementation increased SCFA levels under HFD conditions. Associations were also observed between specific microbial taxa and SCFAs, including positive associations of *Akkermansia* with propionate and butyrate in HFD+RMD mice. SCFAs generated through microbial fermentation can influence host metabolic processes [31], and changes in acetate-related microbial metabolism have been reported in HFD-associated dysbiosis [32]. However, the present study did not directly determine whether microbiota-derived SCFAs contributed to the skeletal muscle phenotype or influenced PI3K/Akt signaling. Therefore, the microbiota, SCFA, and muscle gene-expression findings should be regarded as coordinated associations rather than evidence of an SCFA-mediated signaling mechanism.

In conclusion, RMD supplementation attenuated skeletal muscle atrophy and improved muscle function in aged mice under both SCD and HFD conditions. These effects were accompanied by increased expression of anabolic- and myogenesis-related genes and by alterations in gut microbiota composition and fecal SCFA profiles that differed between dietary conditions. The improvement in muscle outcomes under SCD conditions without a significant reduction in whole-body fat mass suggests that reduced adiposity alone does not explain the muscle benefits associated with RMD. Nevertheless, causal involvement of the gut microbiota or microbial metabolites remains to be established. Future studies using protein-level signaling analyses together with direct manipulation of the gut microbiota or its metabolites will be required to define the mechanisms linking RMD supplementation to skeletal muscle outcomes.

## Supplemental Materials

Supplementary data for this paper are available on-line only at http://jmb.or.kr.
