# Supplementary material for: Dietary Resistant Maltodextrin Attenuates Skeletal Muscle Atrophy in Association with Modulation of the Microbiota–Gut–Muscle Axis in Aged Mice
Source: J Microbiol Biotechnol. 2026 Sep 2;36:e2606037. doi: 10.4014/jmb.2606.06037 (PMC13555004; doi:10.4014/jmb.2606.06037)

J. Microbiol. Biotechnol. 2026. 36: e2606037

https://doi.org/10.4014/jmb.2606.06037

Table. S1. Primer sequence of thermogenesis in adipocyte tissue

Target gene	Forward (5′–3′)	Reverse (5′–3′)
AMPK	TTTGCCTAGAATCCCCACAG	TAAGGAGCCCAGAAAACAGC
SIRT	CTGTTTCCTGTGGGATACCTGACT	ATCGAACATGGCTTGAGGATCT
PGC-1α	CGGAAATCATATCCAACCAG	TGAGGACCGCTAGCAAGTTTG
CEBP/β	ACCGGGTTTCGGGACTTGA	GTTGCGTAGTCCCGTGTCCA
PRDM16	CCCCACATTCCGCTGTGAT	CTCGCAATCCTTGCACTCA
UCP1	CCTGCCTCTCTCGGAAACAA	GTAGCGGGGTTTGATCCCAT
β-actin	AGCTTACTGCTCTGGCTCCTAGC	ACTCATCGTACTCCTGCTTGCT

Table. S2. Primer sequence of mitochondrial biogenesis and muscle differentiation in skeletal muscle.

Target gene	Forward (5′–3′)	Reverse (5′–3′)
Pi3K	CTGGGGGACATACTGACTGT	GTTCCTGGAAAGTCTCCCCTC
Akt	ACTCATTCCAGACCCACGAC	CCGGTACACCACGTTCTTCT
mTOR	ATTCAATCCATAGCCCCGTC	TGCATCACTCGTTCATCCTG
S6K1	TGAGTCAAGCCTTGGTCGAG	AAGAGTCGAGAGAGACGCCC
Myf5	AACCAGAGACTCCCCAAGGT	AGCTGGACACGGAGCTTTTA
MyoD	TGGGATATGGAGCTTCTATCGC	GGTGAGTCGAAACACGGATCAT
MEF2	CAGGCGCTATGGGTCATCTG	GCTACTTGGATTGCTGAACTGC
Myogenin	AGCATCACGGTGGAGGATATG	CAGTTGGGCATGGTTTCGT
FNDC5	GAGGTGCTGATCATCGTCGT	GAGCAAGCACTGAAAGGGTTT
LIF	CAAGAATCAACTGGCACAGC	AGTGGGGTTCAGGACCTTCT
Metrnl	CTGGAGCAGGGAGGCTTATTT	GGACAACAAAGTCACTGGTACAG
β-actin	AGCTTACTGCTCTGGCTCCTAGC	ACTCATCGTACTCCTGCTTGCT

Fig. S1. Analysis of gut bacterial diversity between HFD and HFD+RMD groups.

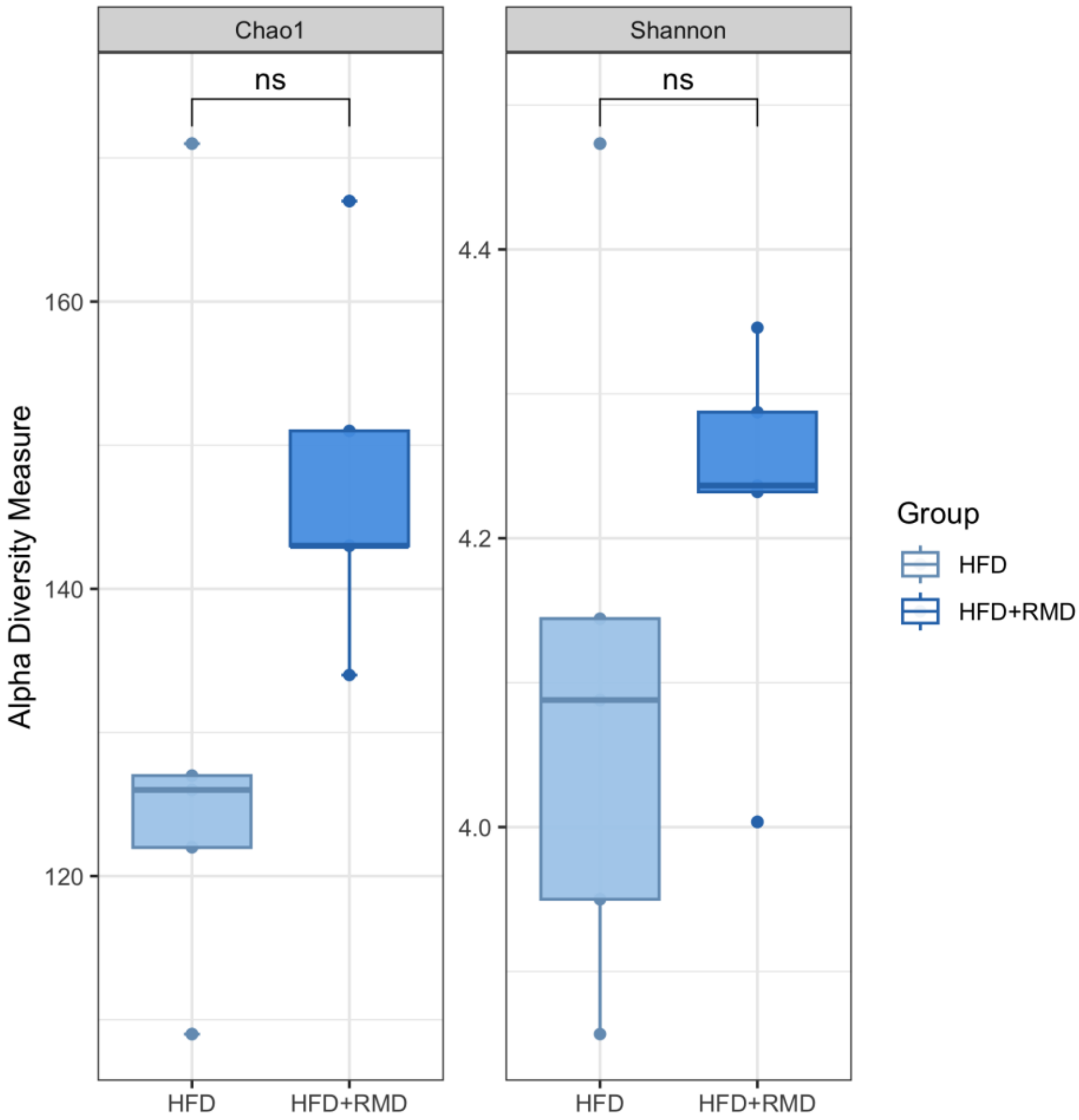

Supplement: Supplementary file 1 [file jmb-36-e2606037-supple.pdf]
